# Bioresorbable High-Strength HA/PLLA Composites for Internal Fracture Fixation

**DOI:** 10.3390/molecules30091889

**Published:** 2025-04-23

**Authors:** Jie Liu, Mingtao Sun, Yipeng He, Weixia Yan, Muhuo Yu, Keqing Han

**Affiliations:** 1 State Key Laboratory of Advanced Fiber Materials, College of Materials Science and Engineering, Donghua University, Shanghai 201620, China; 2 Analysis and Testing Center, Donghua University, Shanghai 201620, China

**Keywords:** HA/PLLA composite, internal fractures fixation, pressure-induced flow, processing modification, mechanical properties

## Abstract

In modern surgery, the internal fixation plates fabricated from hydroxyapatite/poly(L-lactide) (HA/PLLA) composites encounter clinical limitations in fracture treatment due to their inadequate mechanical properties. In this work, pressure-induced flow (PIF) technique is employed to address this limitation. Under optimal processing conditions (140 °C and 250 MPa), the HA/PLLA composites exhibit an impressive flexural strength of 199.2 MPa, which is comparable to that of human cortical bone, the strongest bone tissue in the body. The tensile strength and the notched Izod impact strength are close to 84.2 MPa and 16.7 kJ/m^2^, respectively. Meanwhile, the HA/PLLA composites develop multi-level stacked crystal layers during PIF processing, accompanied by increases in crystallinity (53.1%), crystal orientation (81.6%) and glass transition temperature (78.8 °C). After 2 months of in vitro degradation, the HA/PLLA composites processed by the PIF technique still maintain considerable flexural strength (135.3 MPa). The excellent mechanical properties of HA/PLLA composites processed by PIF technique expand their potential as an internal fixation plate.

## 1. Introduction

In contemporary life, internal fixation plates are routinely employed in surgery to facilitate and expedite fractured patient recovery and functional rehabilitation [1,2]. Conventional metallic fixation plates are limited by several drawbacks, including stress-shielding effects, potential metal ion release, and the necessity for secondary surgical removal [3,4]. Poly(L-lactide) (PLLA), a semi-crystalline biopolymer, has emerged as a particularly promising candidate for internal fixation plate applications, and is distinguished by its unique capacity for complete bioresorbability in the human body and excellent biomimetic properties that closely resemble natural bone tissue [5,6]. The internal fixation plate made of PLLA perfectly overcomes the shortcomings of metal-based materials. However, biological experiments have found that the acidic substances produced by PLLA during degradation induce tissue inflammation, which is not conducive to the recovery of fracture patients [7,8]. Hydroxyapatite (HA), as a calcium phosphate bioceramic with the chemical composition Ca_10_(PO_4_)_6_(OH)_2_, has attracted significant attention as a prominent biomaterial due to its structural similarity to the mineral phase of natural bone, coupled with its exceptional biological properties including biocompatibility, osteoinductivity, osteoconductivity, and inherent bioactivity [9,10]. Unfortunately, the mechanical strength of HA is quite low, making it unsuitable for use as a standalone fixation plate [11]. Currently, no single biomaterial has been demonstrated to comprehensively meet all essential requirements for fracture fixation in clinical applications [12]. Interestingly, the incorporation of HA as a functional filler into PLLA to prepare HA/PLLA composites can maintain the inherent biological characteristics of both components while effectively mitigating inflammatory responses caused by PLLA degradation products [13,14,15]. A large number of biological experiments have proven that the HA content is in the range of 5% to 40%, and the HA/PLLA composite can effectively promote osteoblast adhesion and induce differentiation to form bone tissue [16,17,18]. The osteoinductive capacity of the HA/PLLA composite exhibits a positive correlation with HA content; however, the increase in HA content adversely affects the mechanical strength, demonstrating a distinct trade-off between biological functionality and structural performance [19,20]. Other biomaterials employed in the fabrication of absorbable internal fixation plates are presented in Table 1.

At present, numerous bone fracture fixation products fabricated from HA/PLLA composites have obtained approval from the U.S. Food and Drug Administration (FDA), and have been successfully commercialized in the market [21]. Regrettably, these products have not gained widespread adoption in clinical practice, with one of the primary contributing factors being their insufficient mechanical strength for fracture fixation [22]. According to the ASTM F2502-17 standard [23], flexural strength is regarded as the most critical mechanical criterion for internal fixation plates. Currently, various methods were employed to improve the mechanical properties of materials, including fiber incorporation, chemical copolymerization, and processing modification [24,25,26]. Akindoyo et al. [27] incorporated glass fiber into the HA/PLLA composites, and the results indicated that the tensile strength and impact strength increased by 23% and 42%, respectively. Nevertheless, the non-biodegradable nature of glass fiber restricted their utilization in the field of bone fixation applications. While chemical copolymerization demonstrates distinctive effectiveness in toughening, its capacity to enhance strength remains relatively constrained [28]. Processing modification, as a physical method, can greatly improve the strength and toughness of materials and maintain their original biological properties [29]. Pressure-induced flow (PIF) molding is a process modification technique that alters the microstructure of both crystalline and amorphous phases within semi-crystalline polymers. Lu et al. [30] applied the PIF technique to carbon fiber/polyetheretherketone (CF/PEEK) composites, and the results revealed that the flexural strength increased from 266.4 to 382.0 MPa, showing an enhancement of approximately 43%.

In this work, HA/PLLA composites containing 10% HA were selected, which could ensure the composites’ excellent capability to induce osteogenic differentiation, but also maintain the initial mechanical strength before reinforcement. The PIF technique was further employed to focus on enhancing the flexural strength of HA/PLLA composites. The enhancement mechanism of the PIF technique on the mechanical properties of the HA/PLLA composites was investigated through microscopic morphology observation, crystallization analysis, and thermal analysis. An in vitro degradation experiment of HA/PLLA composites was also performed.

## 2. Results and Discussion

### 2.1. Mechanical Properties of the HA/PLLA Composites

**Figure 1:**
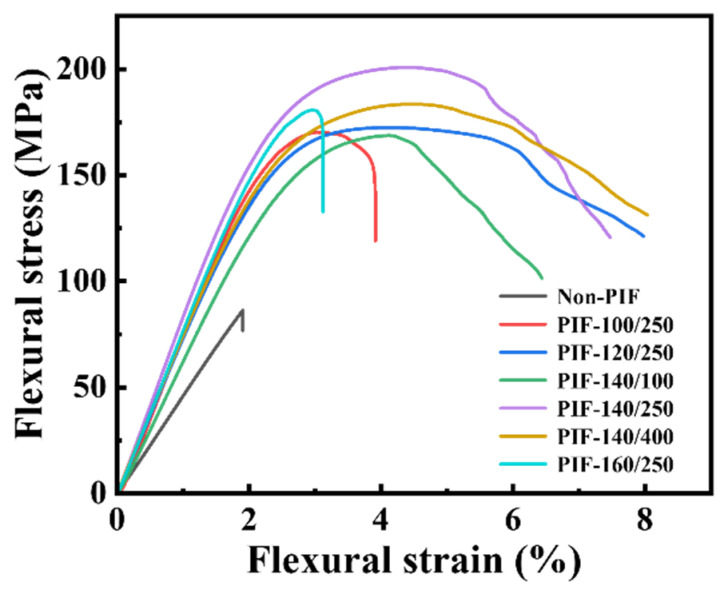
Flexural stress–strain curves of non-PIF and PIF processed HA/PLLA composites.

The typical flexural stress–strain curves of the HA/PLLA composites are shown in Figure 1. The flexural toughness of the HA/PLLA composites was significantly enhanced through PIF processing, as evidenced by their fracture modes. The fracture process of Non-PIF displayed a characteristic brittle fracture behavior, with the stress abruptly dropping to zero upon reaching a maximum value. In contrast, PIF-processed samples exhibited progressive stress reduction after reaching maximum stress, and were accompanied by the development of distinct plateau regions, which were particularly evident for PIF-120/250, PIF-140/250, and PIF-140/400, showing characteristics of ductile fracture.

**Figure 2:**
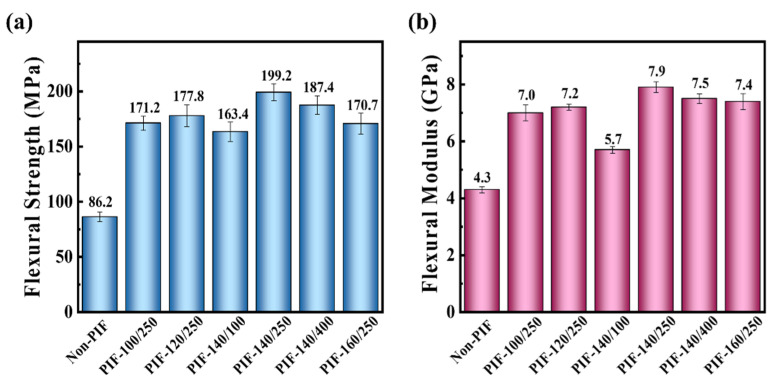
(**a**) Flexural strength and (**b**) flexural modulus of non-PIF and PIF-processed HA/PLLA composites.

Figure 2 presents the flexural strength and flexural modulus of the HA/PLLA composites. The flexural strength of the Non-PIF was 86.2 MPa, and flexural modulus was 4.3 GPa. Compared to the Non-PIF, PIF-140/250 demonstrated a significant improvement in flexural performance, exhibiting a flexural strength of 199.2 MPa and a flexural modulus of 7.9 GPa, which corresponded to increases of 131% and 84%, respectively. The flexural strength of the PIF-140/250 sample is comparable to human cortical bone (200 MPa) [31] and suitable for internal fixation plates. However, both the flexural strength and flexural modulus of HA/PLLA composites showed a decreasing trend when the processing temperature was above 140 °C or the pressure exceeded 250 MPa. This may be attributed to the formation of a continuous crystalline phase resulting from the overcrystallization at high temperature [26], which subsequently impaired the crack deflection. Excessive PIF pressure led to the rupture of crystalline structure, thereby resulting in a reduction in flexural properties.

Considering the flexural strength requirements for internal fracture fixation materials, only the tensile and impact properties of PIF-140/250 were tested, as shown in Table 2. The tensile strength of PIF-140/250 reached 84.2 MPa, exhibiting a 138% enhancement compared to the Non-PIF (35.3 MPa). Significantly, the notched Izod impact strength increased from 3.4 to 16.7 kJ/m^2^, with a 391% increase compared to the non-PIF composite. Conventionally, the improvement in impact toughness is accompanied by a decrease in tensile strength, and vice versa. The simultaneous enhancement of both tensile strength and impact toughness was successfully achieved through PIF processing, which was a signal of a change in the microstructure of the HA/PLLA composites.

### 2.2. Microstructure of the HA/PLLA Composites

**Figure 3:**
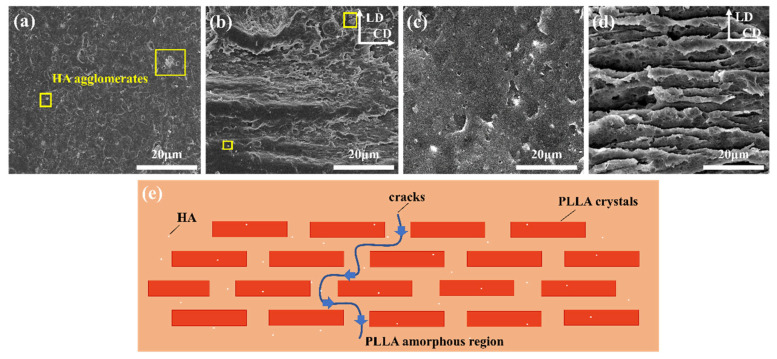
SEM images of flexural fracture surfaces: (**a**) non-PIF sample and (**b**) PIF-140/250. After immersion in NaOH/methanol solution: (**c**) non-PIF sample and (**d**) PIF-140/250 (LD and CD represent the loading direction and the constraint direction, respectively). (**e**) Schematic diagram of crack deflection.

The microstructure can be observed by SEM on the flexural fracture surfaces of HA/PLLA composites, as shown in Figure 3a,b. An aggregation phenomenon of HA was observed in the non-PIF sample and PIF-140/250, due to the poor compatibility between PLLA and HA [16]. After PIF processing, the PLLA matrix produced a solid flow orientation under the combined action of temperature and pressure. To further trace the orientation microstructure of the PLLA matrix more clearly, an NaOH/methanol solution was used to dissolve the HA on the fracture surface while etching the PLLA amorphous region, as shown in Figure 3c,d. Compared with the non-PIF sample, PIF-140/250 had obvious multi-level stacked crystal layers. It is well known that crack propagation preferentially occurs in the amorphous phase since the strength of the crystalline regions is higher than that of the amorphous regions [29]. However, when the fracture behavior occurred, crack propagation could be impeded effectively by the multi-level stacked crystal layers. Figure 3e illustrates a schematic diagram of the layer structure of the HA/PLLA composites processed by PIF technique. The slippage and pull-out of the crystal layer significantly increased the damage energy, leading to the continuous crack deflection within the amorphous regions, which ultimately contributed to the impressive mechanical properties of the PIF-140/250.

### 2.3. Formation of Multi-Level Stacked Crystal Layers

**Figure 4:**
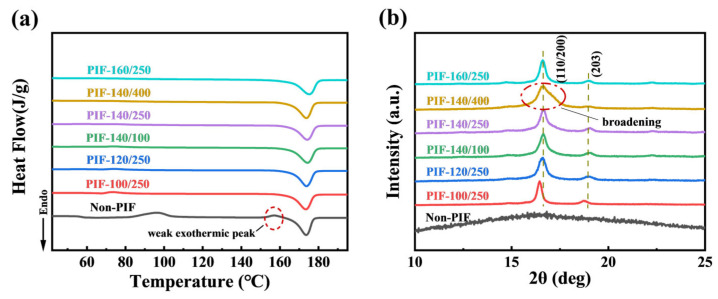
(**a**) DSC heating curves and (**b**) WAXD patterns of non-PIF and PIF-processed HA/PLLA composites.

Figure 4a presents the DSC heating curves of non-PIF and PIF-processed HA/PLLA composites. The thermal parameters derived from Figure 4a are listed in Table 3. The non-PIF sample had an obvious cold crystallization peak and a melting peak, and notably, a weak exothermic peak was observed. During the DSC heating scan, cold crystallization resulted in the formation of numerous α’ crystals, accompanied by the transformation from α’ crystals to α crystals above 120 °C, generating weak exothermic peaks [32]. After PIF processing, all samples only exhibited a distinct endothermic melting peak in the DSC thermogram, suggesting an increase in crystallinity, which was attributed to the synergistic effects of cold crystallization and strain-induced crystallization during the PIF processing. The disappearance of the weak exothermic peak for the HA/PLLA composites PIF processed above 120 °C was due to the fact that the majority of α’-to-α crystal transformation occurred during PIF processing. Differently, no significant weak melting peaks were observed in the sample processed at 100 °C, as the α’-to-α crystal transition was completed at a lower temperature compared to the non-PIF sample, resulting from the markedly reduced cold crystallization effect during the DSC heating scan. Among all samples, the PIF-160/250 had the highest crystallinity degree of 60.4% and the highest melting temperature (T_m_). This may be because the strain-induced crystallization of PLLA was more pronounced, and the thickness of the lamellae of the original crystals was further increased at the combination of high temperature (160 °C, near the T_m_ of PLLA) and pressure [33].

WAXD patterns of non-PIF and PIF-processed HA/PLLA composites are shown in Figure 4b. Within the 2θ range of 10° to 25°, the characteristic peaks of PLLA were observed, without the diffraction peaks corresponding to HA. The WAXD pattern of the non-PIF sample was dominated by amorphous scattering peaks, showing the low crystallinity of PLLA. HA/PLLA composites processed by the PIF technique exhibited distinct diffraction peaks at 2θ ≈ 16.7° and 2θ ≈ 19.0°, corresponding to (110/200) and (203) crystal planes, respectively [34]. Obviously, the diffraction peak of the (110/200) crystal plane shifted to a large angle for the HA/PLLA composites PIF processed above 120 °C compared with the sample at 100 °C. The shift was caused by the transformation from α’ crystal to α crystal. The crystal lattice of α’ crystal exhibits a greater interplanar spacing compared to that of α crystals [32]. In addition, the diffraction peak of the (110/200) crystal plane widened obviously when the pressure was 400 MPa, which was due to the fact that the application of excessive pressure during PIF processing led to the crystal fracture and consequently grain size reduction [35].

**Figure 5:**
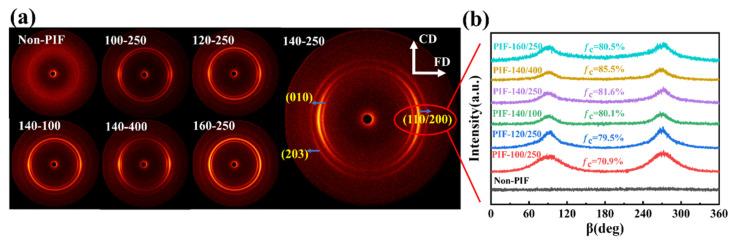
(**a**) 2D-WAXD patterns and (**b**) azimuthal integration curves for the (110/200) crystal plane of non-PIF and PIF-processed HA/PLLA composites. (FD represents the flow direction).

Figure 5a presents the 2D-WAXD patterns of HA/PLLA composites. The non-PIF sample exhibited uniform and blurred diffraction rings, indicating that the PLLA matrix was in an isotropic state. After PIF processing, all samples showed strong diffraction rings and degraded into equatorial arcs, with lamellae rotating from LD to FD [29]. In order to further analyze the change in the orientation of the crystalline region, azimuthal integration curves for the (110/200) crystal plane were obtained from the integral of the 2D-WAXD images, as shown in Figure 5b. No obvious peaks were observed for the non-PIF sample, while distinguishable peaks appeared after PIF processing. The narrower the half-height width of the peak, the higher the degree of orientation in the crystalline region [36,37]. For all PIF-processed samples, the oriented crystals consisted of two parts, namely strain-induced crystals and deformed or rotated primitive crystals. At a processing temperature of 140 °C, the crystallite orientation factor (*f*_c_) of PIF-processed samples increased with an increase in PIF processing pressure. The *f*_c_ of PIF-140/400 reached a maximum of 85.5%, which was related to the fact that the high pressure was in favor of the molecular chains sliding and regularly arranging along the flow direction. At a processing pressure of 250 MPa, the *f*_c_ of PIF-processed samples decreased when the processing temperature exceeded 140 °C. This may be due to the excessive activation of molecular chains induced by elevated temperatures, which facilitated the relaxation and de-orientation of the oriented molecular chains upon pressure release.

**Figure 6:**
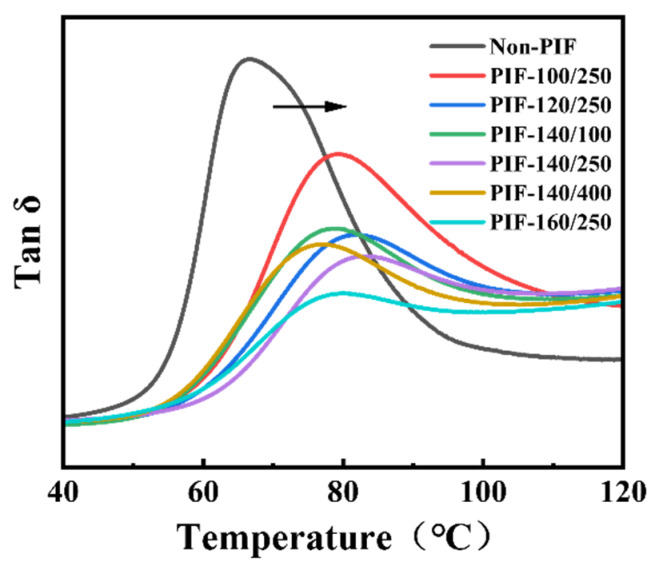
Tan δ curves of non-PIF and PIF-processed HA/PLLA composites.

The tan δ curves of non-PIF and PIF-processed HA/PLLA composites as a function of temperature are illustrated in Figure 6. The glass transition temperature (T_g_) of all PIF-processed samples determined by the peak temperature of tan δ curves dramatically shifted to higher temperatures compared to the non-PIF sample, which was consistent with the DSC results. PIF processing could reduce the free volume required for segment movement, which was hindered and required higher activation energy in the PIF-processed samples. In addition, the height of the tan δ peak also decreased significantly after PIF processing, which was attributed to the significant increase in PLLA crystallinity degree and the tight molecular stacking under the combined effects of pressure and temperature.

Based on the above analysis, it could be inferred that the formation of these unique multi-level stacked crystal layers involved three processes: (1) a large number of crystals were generated through cold crystallization and strain-induced crystallization; (2) the original and newly formed lamellae organized into crystal layers via slippage and rotation; and (3) the formed crystal layers and amorphous phases were compressed under pressure, leading to tight molecular stacking. It is important to note that these three processes occur at the same time.

### 2.4. In Vitro Degradation Property of the HA/PLLA Composites

**Figure 7:**
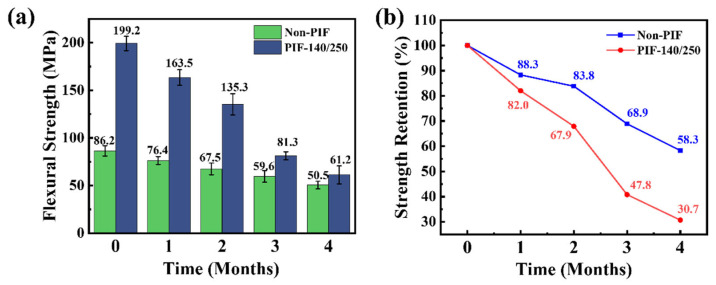
(**a**) Flexural strength and (**b**) strength retention of non-PIF and PIF-140/250 samples during various in vitro degradation cycles.

Generally, the healing time for human bones is approximately 2 to 4 months, with variations depending on individual differences and different bone tissues [38,39]. An ideal bone fixation plate should exhibit a gradual strength reduction that corresponds to the healing process, effectively preventing stress shielding while stimulating proper fracture site recovery [40]. The flexural strength of the HA/PLLA composite subjected to various degradation cycles is illustrated in Figure 7a. After 2 months of in vitro degradation, the PIF-140/250 sample still retained extremely high flexural strength (135.3 MPa) compared to the non-PIF sample (67.5 MPa), indicating the good fixation ability. However, the flexural strength of PIF-140/250 decreased significantly faster than the non-PIF sample during the degradation period, as observed from Figure 7b. After 4 months of in vitro degradation, the strength retention rate of the PIF-140/250 sample was only 30.7%, which was much lower than that of the non-PIF sample (58.3%). As we know, the hydrolytic degradation primarily initiates in the amorphous regions of PLLA [41]. Similarly, for PIF-processed samples, H_2_O tended to diffuse through the amorphous regions between the crystal layers, which resulted from the deflection and fragmentation of lamellar crystals into fine grains that subsequently realigned, with tie molecules connecting these fine grains. It seemed to provide a more convenient path for the diffusion of H_2_O compared with the isotropic non-PIF sample, while being conducive to increased crack propagation and enhanced toughness. As a result, PIF-140/250 samples showed a more rapid decline in flexural strength, yet maintained a higher flexural strength than the non-PIF sample after the same duration of in vitro degradation.

## 3. Materials and Methods

### 3.1. Materials

PLLA (Luminy^®^ L175, optical purity ≥ 99%) was purchased from Total Corbion (Amsterdam, The Netherlands). HA (sphere, average diameter: 200 nm) was supplied by Nanjing Emperor Nano Materials Co. (Nanjing, China).

### 3.2. Sample Preparation

PLLA and HA were subjected to vacuum drying at temperatures of 80 °C and 105 °C for 24 h and 12 h, respectively. The dried PLLA and HA were premixed in a 90/10 mass ratio, and then melt blended and injection molded at 200 °C using a combined micro twin-screw extruder and micro-injection molding machine (DHE-15 and CDM-15, Shanghai Changkai Elec-tromechanical Technology Co., Shanghai, China). The mold temperature was room temperature, and the holding pressure time was 10 s.

**Figure 8:**
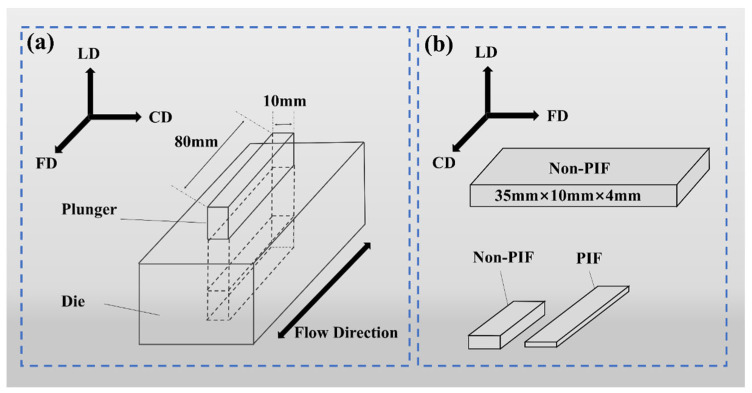
(**a**) Schematic diagram of PIF processing mold, and (**b**) appearance of the sample before and after PIF processing.

The mold for PIF processing is illustrated in Figure 8a. In advance, the mold was preheated to the processing temperature. Subsequently, the sample was transferred to the mold and heated for 10 min. Finally, the pressure was applied for 5 min. The appearance of the sample before and after PIF processing is shown in Figure 8b. The unprocessed sample is designated “Non-PIF”, while the PIF samples are labeled “PIF-temperature/pressure”.

### 3.3. Characterization

#### 3.3.1. Mechanical Properties Tests

Flexural tests were conducted using a universal testing machine (ETM 105D, Wance, Shenzhen, China) in accordance with ISO 178: 2019 [42].

Tensile tests were conducted on a universal testing machine (ETM 105D, Wance, Shenzhen, China) at a constant crosshead speed of 2.0 mm/min in accordance with ISO 527-2:2012 [43].

Notched Izod impact tests were performed on a pendulum impact testing machine (TSP501J, Shenzhen, Wance, China) according to ISO 180:2023 [44].

#### 3.3.2. Scanning Electron Microscopy (SEM)

The fracture surface morphology of the samples was characterized by scanning electron microscopy (JSM-IT300, JEOL, Akishima, Japan) at 12.0 kV. The fracture surfaces were platinum-coated to ensure clear imaging.

The crystalline region of PLLA has been shown to possess significantly higher hydrolysis resistance compared to the amorphous region [41]. To more clearly visualize the microstructure of the crystalline region, the non-PIF and 140–250 samples were etched using a NaOH/methanol solution (0.025 mol/L NaOH: methanol = 1:2 by volume) at 50 °C for 2 h. Additionally, etching treatment also promoted the dissolution of surface HA [45]. After etching, the samples were dried in a desiccator for 24 h and subsequently removed for morphological observation.

#### 3.3.3. Differential Scanning Calorimetry (DSC)

Thermal properties of the HA/PLLA composite were characterized using differential scanning calorimetry (DSC 2500, TA Instruments, New Castle, DE, USA). The measurements were conducted under a nitrogen atmosphere at a heating rate of 10 °C/min over a temperature range of 30–200 °C. The calculation of the crystallinity (X_c_) was performed in accordance with the following formula:

(1) $$X_{C}=\frac{\Delta Hₘ-\Delta Hc}{\Delta H^{0}ₘ\times 0.9}\times 100\%$$

where ΔHₘ and ΔH_c_ denote the enthalpy associated with the melting and cold crystallization processes of PLLA, respectively. ΔH⁰ₘ represents the theoretical melting enthalpy of 100% crystalline PLLA, about 93.7 J/g [46].

#### 3.3.4. Wide-Angle X-Ray Diffraction (WAXD)

The X-ray diffractometer (D8 Advance, Bruker, Munich, Germany) equipped with a Ni-filtered Cu Kα radiation source at a wavelength of 0.154 nm was used to characterize the crystal structure. Measurements were performed at 40 kV and 150 mA, with scan angles from 5° to 30° and a scanning rate of 2°/min.

Two-dimensional wide-angle X-ray diffraction (2D-WAXD) measurements were performed using a D8 Discover diffractometer (Bruker, Munich, Germany) operated at 40 kV and 40 mA. The instrument was equipped with a radiation source emitting X-rays at a wavelength of 0.154 nm. Diffraction patterns were acquired using a two-dimensional VANTEC-500 detector (VANTEC Corporation, Yokohama, Japan) with an exposure time of 90 s, while maintaining a sample-to-detector distance of 10 mm. After azimuthal integration of the 2D-WAXD diffraction ring, the crystallite orientation factor (*f*_c_) was calculated by the following formula:

(2) $$f_{C}=\frac{360^{{}^{\circ}}-\sum H_{i}}{360^{{}^{\circ}}}$$

where H_i_ represents the width at the half maximum of the i-th diffraction peak obtained from the azimuthal intensity profile in 2D-WAXD analysis.

#### 3.3.5. Dynamic Mechanical Analysis (DMA)

Dynamic mechanical analysis was performed using a Discovery DMA 850 instrument (TA Instruments, New Castle, DE, USA) operated in single-cantilever mode. The temperature was ramped up from 30 °C to 140 °C at a heating rate of 3 °C/min, while maintaining an oscillation frequency of 1 Hz and an amplitude of 10 μm under controlled strain conditions.

#### 3.3.6. In Vitro Degradation Test

Phosphate buffer saline (PBS) solution was used for the in vitro degradation test. Samples were individually sealed in screw-cap containers with a PBS solution volume (mL) to sample mass (g) ratio exceeding 30:1. The degradation process was performed at 37 °C for predetermined immersion periods of 0, 1, 2, 3, and 4 months.

## 4. Conclusions

Herein, bioresorbable high-strength HA/PLLA composites were prepared through PIF processing. By optimizing the processing parameters, PIF-140/250 achieved a maximum flexural strength of 199.2 MPa, representing a 131% increase compared to the non-PIF sample (86.2 MPa). The tensile strength and the notched Izod impact strength of PIF-140/250 reached 84.2 MPa and 16.7 kJ/m^2^, respectively. As revealed by SEM images of flexural fracture surfaces, PIF-140/250 formed multi-stacked crystal layers. This unique structure significantly promoted increased crack propagation, thereby enhancing energy dissipation, which consequently led to the improvements in both the strength and toughness of the HA/PLLA composites. The X_c_ of PIF-140/250 was close to 53.1% owing to the synergistic effect of cold crystallization and strain-induced crystallization, while the *f*_c_ increased to 81.6%. In the in vitro degradation experiment at 37 °C, the flexural strength of PIF-140/250 was maintained above 135.3 MPa for 2 months, indicating its potential as an internal fixation plate. However, PIF-140/250 showed a more pronounced reduction in flexural strength, during the in vitro degradation. In summary, this work demonstrates the development of bioresorbable high-strength HA/PLLA composites suitable for bone internal fixation applications.

## Figures and Tables

**Table 1:** Bioresorbable materials applied to internal fixation plates.

Classification	Name	Peculiarity
bio-polyester	poly(D,L-lactic acid)	amorphous, degraded quickly
bio-polyester	poly(lactic-co-glycolic acid)	low strength, degraded quickly
bio-ceramics	tricalcium phosphate (Ca_3_(PO_4_)_2_)	osteoinductivity

**Table 2:** The partial mechanical properties of the non-PIF sample and PIF-140/250.

Sample	Tensile Strength (MPa)	Notched Izod Impact Strength (kJ/m^2^)
Non-PIF	35.3 (±4.3)	3.4 (±0.1)
PIF-140/250	84.2 (±6.7)	16.7 (±1.6)

**Table 3:** Thermal parameters of non-PIF and PIF-processed HA/PLLA composites.

Samples	T_g_ (℃)	ΔH_c_ (J/g)	ΔH_m_ (J/g)	T_m_ (℃)	X_c_ (%)
Non-PIF	57.2	21.7	41.1	173.4	23.1
PIF-100/250	77.2	3.4	43.7	173.4	47.8
PIF-120/250	77.5	1.5	43.8	173.8	50.2
PIF-140/100	64.5	0	42.4	174.4	50.3
PIF-140/250	78.8	0	44.7	174.3	53.1
PIF-140/400	63.8	0	44.1	173.7	52.4
PIF-160/250	77.3	0	50.9	175.1	60.4

## Data Availability

The original contributions presented in this study are included in the article. Further inquiries can be directed to the corresponding author.
